# Medication errors: a prospective cohort study of hand-written and computerised physician order entry in the intensive care unit

**DOI:** 10.1186/cc3793

**Published:** 2005-08-08

**Authors:** Rob Shulman, Mervyn Singer, John Goldstone, Geoff Bellingan

**Affiliations:** 1 ICU Pharmacist, Pharmacy Department, University College London Hospitals, Middlesex Hospital, London, UK; 2 Consultant, Critical Care Directorate and Professor, Department of Medicine and Wolfson Institute of Biomedical Research, University College London, Middlesex Hospital, London, UK; 3 Consultant, Intensive Care and Anaesthetics Department, University College London Hospitals, Middlesex Hospital, London, UK; 4 Consultant and Clinical Director, Critical Care Directorate, University College London Hospitals, Middlesex Hospital, London, UK

## Abstract

**Introduction:**

The study aimed to compare the impact of computerised physician order entry (CPOE) without decision support with hand-written prescribing (HWP) on the frequency, type and outcome of medication errors (MEs) in the intensive care unit.

**Methods:**

Details of MEs were collected before, and at several time points after, the change from HWP to CPOE. The study was conducted in a London teaching hospital's 22-bedded general ICU. The sampling periods were 28 weeks before and 2, 10, 25 and 37 weeks after introduction of CPOE. The unit pharmacist prospectively recorded details of MEs and the total number of drugs prescribed daily during the data collection periods, during the course of his normal chart review.

**Results:**

The total proportion of MEs was significantly lower with CPOE (117 errors from 2429 prescriptions, 4.8%) than with HWP (69 errors from 1036 prescriptions, 6.7%) (p < 0.04). The proportion of errors reduced with time following the introduction of CPOE (p < 0.001). Two errors with CPOE led to patient harm requiring an increase in length of stay and, if administered, three prescriptions with CPOE could potentially have led to permanent harm or death. Differences in the types of error between systems were noted. There was a reduction in major/moderate patient outcomes with CPOE when non-intercepted and intercepted errors were combined (p = 0.01). The mean baseline APACHE II score did not differ significantly between the HWP and the CPOE periods (19.4 versus 20.0, respectively, p = 0.71).

**Conclusion:**

Introduction of CPOE was associated with a reduction in the proportion of MEs and an improvement in the overall patient outcome score (if intercepted errors were included). Moderate and major errors, however, remain a significant concern with CPOE.

## Introduction

Medication errors (MEs) in the intensive care unit (ICU) are common and can arise from a number of causes. A large study from two tertiary care hospitals reported the error rate was highest in medical ICUs (19.4 per 100 patient days), particularly at the prescribing stage, which accounted for 56% of errors detected [1]. The National Health Service Plan in the UK [2] states that 75% of hospitals should have implemented electronic patient record systems by 2004 in order to make information available at the point of need. Computerised physician order entry (CPOE) without decision support may have advantages over hand-written prescribing (HWP) in terms of standardisation, full audit trail, legibility, use of approved names, specification of key data fields such as route of administration, storage and recall of records.

Although the CPOE system recently installed in our ICU has access to our locally produced on-line formulary (which includes local guidelines), IV guide (advising how to safely administer intravenous medications), drug interactions, contraindications and side effects, these are for information only and decision support capability does not exist. Systems with decision support offer the ability to prevent physicians prescribing either a known allergenic drug or a toxic drug dose [3]. It can flag up drug-drug interactions, force compliance with hospital protocols, and can prevent the prescription of certain drugs, thus implementing evidence based medicine [4] and improving clinical practice [5-7]. This prospective study compares HWP with CPOE without decision support, in several ways. We compare the rates and types of MEs and the potential outcome of intercepted and non-intercepted errors.

## Materials and methods

In April 2002, University College Hospitals London ICU introduced the QS 5.6 Clinical Information System (CIS) (GE Healthcare, Anapolis, MD, USA) to the ICU but not on the general wards. The new system was introduced following a program of staff training and HWP was completely changed on a single day. The system used offers a CPOE component but without decision support. Prior to this, hand-written drug charts were used. With both prescribing systems, prescribing was restricted to intensive care medical staff only. To compare both prescribing systems, details of all MEs identified by the ICU clinical pharmacist, in the course of his normal prescription review, were prospectively recorded before the change period and for four reasonably evenly spaced data collection periods after the introduction of the CPOE. The study was designed in advance to collect data over a 70 week time period to enable reliable estimates of error rates. The HWP data collection began on the following dates: 17 September 2001 for 5 days; 24 September 2001 for 4 days. CPOE data collection began on the following dates: 15 April 2002 for 5 days; 10 June 2002 for 2 days; 27 September 2002 for 5 days; and 18 December 2002 for 5 days. CPOE and HWP sample sizes were of different lengths so that an assessment of learning curve could take place. We aimed for each monitoring period to be 5 days. The first two HWP periods were consecutive and thus merged in the results. One period was curtailed due to investigator illness. The ICU medical and nursing staff were unaware that the study was being conducted. Ethical approval was not sought, because at the time audits were not within the remit of the local ethics committee. Prior to introduction of CPOE, local standards of prescribing existed specifying the tenets of good practice, including the avoidance of the use of abbreviations.

An ME was defined to have occurred when a prescribing decision or prescription writing process resulted in either an unintentional significant reduction in the probability of treatment being timely and effective or an unintentional significant increase in the risk of harm when compared with generally accepted practice [8]. During the monitoring period, details of the total number of all prescribed drugs on each day were recorded.

MEs were assessed by type and patient outcome. The type of error was categorised by the pharmacist into groups that best represented the data. A single error could be recorded as several types of error. The total numbers of MEs were also recorded. If a single drug episode was judged to be in error for multiple reasons, it was counted only once for the error rate analysis.

The patient outcome from each error were assigned by the pharmacist and the ICU clinical director, according to an adapted scale [9-11]. Minor errors were classified as those causing no harm or an increase in patient monitoring with no change in vital signs and no harm noted. Moderate errors were classified as those causing an increase in patient monitoring, a change in vital signs but without associated harm or a need for treatment or increased length of stay. Major errors were categorised as those causing permanent harm or death. In this study, intercepted errors (e.g. where an incorrect dose of a drug was prescribed but not administered) were separated from non-intercepted errors (where the patient received the drug). The intercepted errors were scored separately on the basis of their possible impact on the patient, if the prescription had been administered as prescribed.

The chi squared test for trend was used to test whether there was a learning effect over time with CPOE. A chi squared test was used to test for the error rates and outcome comparisons. A two tailed t test was used to compare means of APACHE II score for the HWP and CPOE periods. For this test, as the Levene's test was not significant, equal variance was assumed.

## Results

**Figure 1:**
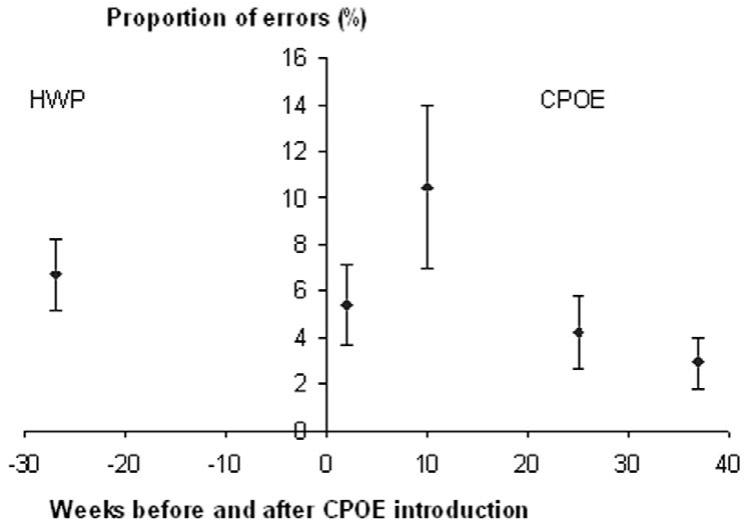
Proportion of medication errors before and after implementation of computerised physician order entry (CPOE) using the Clinical Information System with 95% confidence intervals. Hand-written prescribing (HWP) data collection began on the following dates: 17 September 2001 for 5 days; 24 September 2001 for 4 days (merged with the previous period). CPOE data collection began on the following dates: 15 April 2002 for 5 days; 10 June 2002 for 2 days; 27 September 2002 for 5 days; and 18 December 2002 for 5 days.

The mean Acute Physiology and Chronic Health Evaluation (APACHE) II scores for the HWP (19.4, standard deviation 9.5, n = 56) and CPOE (20.0, standard deviation 8.0, n = 99) periods were not significantly different (p = 0.71). In the study, 134 drug charts with 1036 prescriptions were reviewed in the HWP group and 253 charts with 2429 prescriptions were assessed in the CPOE group. The proportion of MEs for each data collection period are shown in Fig. 1. The proportion of MEs before CPOE was 6.7% (69 errors from 1036 prescriptions) and 4.8% after CPOE introduction (117 errors from 2429 prescriptions) (p < 0.04). Thus, the reduction in the proportion of MEs following the introduction of CPOE was statistically significant. The proportion of MEs with CPOE varied over time after its introduction (p < 0.001). Evidence also indicated the strong linear trend of a declining proportion of MEs over time (p < 0.001). The types of error from the two systems are listed in Table 1. CPOE appeared to be associated with a high number of dosing errors, omission of the required drug and the prescriber's signature. A number of hand-written prescriptions were missing key details, for example, dose, units or frequency. Several incidences were noted with CPOE in which a drug was not prescribed; for example, caspofungin was omitted when a patient previously established on this drug was admitted to the ICU. Although we did not prospectively look for all missed prescriptions, standard care was for the pharmacist to review admissions and note discrepancies between ward and ICU prescriptions. This error occurred during the CPOE prescribing period.

The patient outcome scores are given in Tables 2 and 3. Most of the errors were minor in outcome, although two non-intercepted errors with CPOE led to an increased length of stay or increased monitoring. In the first case, an anuric patient on haemofiltration was prescribed and administered gentamicin 500 mg, which resulted in prolonged toxic levels. In the second case, a unique problem to CPOE occurred when a loading dose of phenytoin was not administered because a stage of prescription activation was not correctly carried out; the computer-generated order for the nurse to administer the drug was not triggered due to poor prescribing practice, leading to the dose being omitted. This resulted in an extended period before seizure control was achieved.

Three intercepted errors with CPOE could have caused permanent harm or death if they had been administered as prescribed. These intercepted errors were not administered to the patient because either the pharmacist intercepted the prescription before administration or the nurse recognised the error. A potentially fatal intercepted error occurred when diamorphine was prescribed electronically using the pull down menus at a dose of 7 mg/kg instead of 7 mg, which could have lead to a 70 times overdose. In a separate case, amphotericin 180 mg once daily was prescribed, when liposomal amphotericin was intended. The doses of these two products are not interchangeable and the high dose prescribed would have been nephrotoxic. In the third case, vancomycin was prescribed 1 g intravenously daily to a patient in renal failure, when the appropriate dose would have been to give 1 g and then to repeat when the plasma levels fell below 10 mg/L. The dose as prescribed would have lead to nephrotoxicity.

There were many cases of minor errors with CPOE that did not cause patient harm but did increase monitoring. With respect to the non-intercepted errors, there was no significant difference between groups (p = 0.51; Table 3). If we include intercepted errors, however, there is a difference due to the increased rate in the HWP group (p = 0.01; Table 3). It is of note that the only major errors encountered were the three major intercepted errors attributed to CPOE. It appears that CPOE was associated with more minor errors that did not cause patient harm but did increase monitoring.

## Discussion

This study was designed to investigate the impact of CPOE, without decision support, on MEs in the critical care setting. The data collected were viewed in terms of proportion of errors, patient outcomes arising from the error and types of error.

The proportion of MEs reduced following the introduction of CPOE. There was also some evidence that a learning curve occurred with CPOE, as the proportion of errors appeared to decline over time. This learning curve could have included improvements made to the system in light of experience, although it is conceivable that the ME rate may have reduced by itself over time. The error rates found were less than those reported in a recent study of prescription errors in UK critical care units [12]. There was no difference in the mean APACHE II score in the HWP and CPOE periods, indicating that it is unlikely that severity of illness differed substantially in the monitored periods.

It was decided to separate the recording of non-intercepted and intercepted errors (where an error was spotted and corrected before having an impact on the patient). The intercepted errors were scored on the basis of what might have occurred if the patient received the medication as prescribed. There was a demonstrated benefit on patient outcome scores with CPOE prescribing when the intercepted errors were combined with the non-intercepted errors. It was reassuring to note that no patients suffered permanent harm or death as a result of any non-intercepted error. Three errors, which all occurred with CPOE, could have led to permanent harm or death had they been administered as prescribed. This CPOE system lacks the ability to effectively deal with drugs with variable dosage regimens such as vancomycin, gentamicin and warfarin. In addition, our impression is that prescribers often prescribed too quickly and made mistakes when using pull-down menus, as seen with the diamorphine error. A lack of product knowledge probably led to the amphotericin error. Prescribers need to develop a thorough, systematic approach to prescribing, similar to that which they employ for diagnosis. This aspect of our findings is in accordance with a recent study that identified that a CPOE system frequently increased the probability of prescribing errors [13].

Most of the errors were defined as 'minor' in outcome and, as such, did not cause the patient harm but, in some cases, may have lead to an increase in monitoring but with no change in vital signs. There were four errors, however, that caused either patient harm or increased monitoring and 34 intercepted errors that could have potentially caused harm had they been administered. The fact that these MEs were rectified before they harmed the patient underlines the value of daily prescription review by an experienced clinical pharmacist [14,15]. In contrast to other views [8], it was decided not to regard abbreviated drug names as errors, because this would have distorted the results in favour of CPOE. In justification of this treatment of the results, no abbreviated drug name led to a patient receiving the wrong drug, but it is regarded as poor prescribing practice as defined by our own prescribing guidelines and national guidelines [16]. CPOE effectively eradicated the use of abbreviations.

The study was not designed or powered to identify differences in the types of errors under the two systems. Future work should be designed to focus on these differences. Omission of key prescription details such as dose, units, frequency and signatures appeared to be much reduced with CPOE, as the computer program did not permit drug entry with missing key data entry fields. Dose errors were still prevalent with CPOE, however, as a result of physicians choosing the wrong drug template, selecting from multiple options, or as a consequence of constructing their own drug prescriptions using pull down menus.

There were also many missed prescribers' signatures with CPOE. This did not affect the patient but, in these cases, nurses administered medication without a legally valid physician order. Although an absent 'signature' with CPOE was regarded as an error, the audit facility of the Clinical Information System did record who prescribed the drug. There were several cases where necessary drugs were not prescribed with CPOE; this was probably not related specifically to the prescribing system.

The categories described were specific to the setting and systems, thus a general taxonomy of medication errors [17] was not used as it was considered that this did not adequately characterise the errors. The categories used here specifically describe the event and general taxonomies were considered to be too broad to provide a specific and useful description of the episode.

During the data collection period, key staff such as consultants, senior nurses and the pharmacist remained the same, so this did not influence the results. Pharmacist attendance at ward rounds has been associated with a reduction in adverse events [15]. In this study the pharmacist attended the ward round throughout the study. No other significant organisational changes occurred during the study period. The only possible changes were the junior medical staff who did change during the study and this may have affected the results. Ideally, the impact of this could be minimised by sampling over a longer period and more frequently, but this was beyond the scope and resources of this study. Alternatively, we could have statistically adjusted for experience level, although this is a difficult issue and has not been attempted by other researchers. Furthermore, the MEs recorded were all proactively identified from the daily pharmacist prescription chart review, and thus did not rely on the notoriously low reporting of multi-disciplinary adverse incident reports. Patient outcome was assessed by the pharmacist and clinical director, who were not blinded to the prescribing system; this could have introduced the potential for bias in the results and is a limitation of the study.

Medical errors are among the leading causes of death in the United States. In its highly publicised report, the Institute of Medicine estimates that between 44,000 and 98,000 Americans die as a result of medical errors each year, with the majority of these errors being preventable [18]. MEs are the leading type of medical error [3]. Previously, in a setting that included general wards and ICUs, a similar type of CPOE was associated with a halving of the rate of non-intercepted MEs [19]; ours is the first study identified that investigates the impact of CPOE on MEs solely in an adult ICU. CPOE is already the subject of considerable interest [20] and has already shown benefits in paediatric medicine [21-23]. A systematic review of the impact of clinical decision support systems (CDSS) [6] has demonstrated statistically significant improvements in antibiotic-associated MEs or adverse drug events and an improvement in theophylline-associated MEs, while several studies have shown non-significant results. CDSS is worthy of future study in the adult ICU in order to build on the experience gained from the limited CDSS system used in a mixed ICU and general ward setting [19].

## Conclusion

These results indicate that the introduction of CPOE, without decision support, in our ICU was associated with a reduced proportion of MEs and improved patient outcome after an error (when non-intercepted and intercepted errors were combined). The limitations of this study and the potential for bias discussed previously must be borne in mind when interpreting these results.

Some of the types of errors appeared to change with CPOE; of particular concern was the finding that all three of the major intercepted errors arose with CPOE. In our study, CPOE clearly reduced the incidence of less major errors but the more serious errors are a genuine concern with this CPOE system. This is not an isolated finding [13] and should be noted by clinical directors as they review the need for CPOE on their units. As clinicians embrace CPOE, they should not make the assumption that CPOE removes errors; in fact, different types of errors emerge. We cannot abdicate our responsibility for ensuring that a prescription is correct in favour of a computer.

## Key messages

• 	This study is the first to compare CPOE and HWP solely in the ICU.

• 	CPOE was associated with a reduced proportion of MEs compared with HWP and this lowered over time.

• 	When intercepted and non-intercepted errors were combined, CPOE was associated with an improvement in the error outcome scoring compared to HWP; however, the three intercepted errors that could have caused permanent harm or death all occurred with CPOE.

• 	The types of error appeared to change with the introduction of CPOE.

• 	The introduction of CPOE without decision support eliminated many minor types of error but introduced new types of error that may be more serious.

## Abbreviations

APACHE = Acute Physiology and Chronic Health Evaluation; CDSS = clinical decision support systems; CPOE = computerised physician order entry; HWP = hand-written prescribing; ICU = intensive care unit; ME = medication error.

## Competing interests

The authors declare that they have no competing interests.

## Authors' contributions

RS conceived the study, collected the data, analysed the results and drafted the article. MS was involved in critically revising the draft. JG made substantial contributions to the data analysis. GB was substantially involved in the analysis, interpretation and drafting the manuscript.

## Figures and Tables

**Table 1:** Types of medication errors before and after implementing CPOE

Error type	HWP (no. of errors and % of total errors)^a^	CPOE (no. of errors and % of total errors)^a^
Drug prescribed on incorrect drug chart section (e.g. continuous IV infusion prescribed on 'when required' part of drug chart)	2 (2.8%)	1 (0.9%)
Drug needed but not given as not prescribed properly	3 (4.2%)	5 (4.3%)
Inappropriate/inadequate additional information on prescription to adequately administer the drug appropriately	8 (11.3%)	12 (10.3%)
Dose/units/frequency omitted on prescription	22 (31%)	1 (0.9%)
Prescription not signed or change not signed/dated	10 (14.1%)	39 (33.3%)
Still wrong next day after pharmacist recommended appropriate correction that was agreed with doctor	0 (0%)	3 (2.6%)
Dose error	12 (16.9%)	31 (26.5%)
Wrong drug prescribed	3 (4.2%)	6 (5.1%)
Incorrect route/unit	5 (7%)	8 (6.8%)
Formulary not followed without reason	3 (4.2%)	1 (0.9%)
Administration not in accordance with prescription	3 (4.2%)	3 (2.6%)
Required drug not prescribed	0 (0%)	7 (6%)
Total	71/1036 prescriptions	117/2429 prescriptions

^a^One episode could be recorded here as being in error for several reasons but was only recorded once in the proportion of error analysis. This explains why the total of hand-written prescribing (HWP) error types stated here is in excess of the total number of errors stated in the results section. CPOE, computerised physician order entry.

**Table 2:** Error outcome categories

Error category	Minor	Moderate	Major
HWP non-intercepted errors	43	0	0
CPOE non-intercepted errors	93	4	0
HWP intercepted errors	7	19	0
CPOE intercepted errors	2	15	3

CPOE, computerised physician order entry; HWP, hand-written prescribing.

**Table 3:** Error outcome category analysis

Error category	None	Minor	Moderate/major	Total
Non-intercepted errors^a^				
HWP	993 (95.9%)	43 (4.2%)	0 (0%)	1036
CPOE	2332 (96.0%)	93 (3.8%)	4 (0.2%)	2429
Non-intercepted plus intercepted errors^b^				
HWP	967 (93.3%)	50 (4.8%)	19 (1.8%)	1036
CPOE	2312 (95.2%)	95 (3.9%)	22 (0.9%)	2429

^a^No significant difference with regard to errors between hand-written prescribing (HWP) and computerised physician order entry (CPOE; p = 0.51).

^b^If we include intercepted errors, there was a significant difference (p = 0.01) due to increased error rate with HWP.
